# Red Blood Cell Distribution Width Is Associated with Carotid Atherosclerosis in People with Type 2 Diabetes

**DOI:** 10.1155/2018/1792760

**Published:** 2018-03-26

**Authors:** J. S. Nam, C. W. Ahn, S. Kang, K. R. Kim, J. S. Park

**Affiliations:** ^1^Department of Internal Medicine, Yonsei University College of Medicine, Seoul, Republic of Korea; ^2^Severance Institute for Vascular and Metabolic Research, Yonsei University College of Medicine, Seoul, Republic of Korea

## Abstract

**Aims:**

Red cell distribution width (RDW) has been shown to be associated with cardiovascular diseases (CVD). The relationship between RDW and carotid intima-media thickness (C-IMT), a marker of subclinical atherosclerosis, has been inconsistent in subjects with cardiovascular risk factors. In this study, we investigated the relationship between RDW and carotid atherosclerosis in people with type 2 diabetes.

**Methods:**

Four hundred sixty-nine people with type 2 diabetes without history of cardiovascular or cerebrovascular diseases were enrolled. Anthropometric measures and various biochemical parameters including RDW were assessed. Ultrasonographic measurement of carotid intima-media thickness was used to evaluate subclinical atherosclerosis.

**Results:**

The participants were stratified into 3 groups according to RDW. The C-IMT increased gradually according to RDW tertiles (lowest, second, highest RDW tertiles; 0.740 ± 0.120, 0.772 ± 0.138, and 0.795 ± 0.139, respectively; *p* < 0.01). Multiple regression analysis and multivariate logistic regression analysis revealed that RDW was associated with C-IMT in people with type 2 diabetes, and it remained significant after control for various cardiovascular risk factors including body mass index, blood pressure, insulin resistance, and smoking status in multivariate logistic regression analysis.

**Conclusion:**

RDW is associated with subclinical atherosclerosis assessed by carotid IMT after control of various covariates in people with type 2 diabetes without cardiovascular or cerebrovascular diseases.

## 1. Introduction

Red cell distribution width (RDW) indicates the size variability of circulating erythrocytes and often reported as a part of the complete blood count for the differential diagnosis of anemia [1, 2]. Recent studies have demonstrated that increased RDW is an independent predictor of overall as well as cardiovascular mortality [3–6]. However, the mechanism underlying this relationship between RDW and cardiovascular disease (CVD) remains unclear.

Ultrasonographic measurement of carotid intima-media thickness (C-IMT) is a relatively simple, noninvasive way to assess subclinical atherosclerosis in high-risk patients. CVD is the most common cause of death in people with type 2 diabetes, and C-IMT has been widely used to predict CVD risk and related outcomes in these people [7–10]. There are few studies that have assessed the relationship between RDW and C-IMT in general as well as high-risk populations. Although some conflicting data exist, several studies have verified the association between RDW and C-IMT among people with cardiovascular risks including hypertension, stroke, and chronic kidney disease [11–16]. However, the association between RDW and C-IMT is not known in people with type 2 diabetes.

In this study, we analyzed the relationship between RDW and subclinical atherosclerosis measured by C-IMT and examined its potential role as a marker carotid atherosclerosis in Koreans with type 2 diabetes without CVD.

## 2. Materials and Methods

### 2.1. Subjects

Four hundred sixty-nine people with type 2 diabetes at the Diabetes Center of Gangnam Severance Hospital, Korea, were enrolled in this cross-sectional study. The subjects were retrospectively recruited from Cohort Study for Clinical Research in Gangnam Severance Hospital. This study is an observational study designed to systemically collect clinical and biochemical information of people with impaired glucose metabolism in the Gangnam area in Seoul, Korea, and to establish a cohort to be followed for the incidence of diabetes among those at prediabetic phase and also diabetic complications. Previously diagnosed diabetes patients based on self-reported responses and newly diagnosed diabetes patients according to the American Diabetes Association criteria were all included. People with concurrent acute illnesses including clinically significant infectious diseases, chronic kidney or hepatic diseases, malignancy, and any systemic hematologic disorders that could affect red blood cells were excluded. Those with prior cardiovascular or cerebrovascular diseases were also excluded. Among the 577 type 2 diabetes patients enrolled in Cohort Study for Clinical Research in Gangnam Severance Hospital between 2013 and 2014, 61 subjects with a history of coronary artery disease or cerebrovascular accident, 22 subjects with chronic kidney disease or chronic hepatitis disease, 4 subjects with cancer, and 2 subjects with acute infection were excluded, and 469 subjects were analyzed. The institutional review board of Yonsei University College of Medicine approved this study protocol, and written informed consent was obtained from all subjects.

### 2.2. Anthropometric Measurements

Body weight and height were measured in the morning, without clothing and shoes, and body mass index (BMI) was calculated by dividing the weight (kg) by the square of the height (m^2^). Systolic and diastolic blood pressures were measured by an experienced technician by placing the left arm at heart level after a five-minute rest using EASY X 800 (Jawon Medical Co. Ltd, Seoul, Korea). Current smoking was defined as having smoked cigarettes regularly over the previous 6 months.

### 2.3. Biochemical Parameters

Blood samples were taken from all subjects after an overnight fast. Standard methods were used for complete blood count and biochemistry. Fasting plasma glucose (FPG), total cholesterol (TC), high-density lipoprotein cholesterol (HDL-C), and triglycerides (TG) levels were determined using enzymatic methods with a Hitachi 7600-120 automated chemistry analyzer (Hitachi, Tokyo, Japan). Low-density lipoprotein cholesterol (LDL-C) was calculated according to the Friedewald formula. Hemoglobin A1c (HbA1c) was determined by high-performance liquid chromatography (Variant II, Bio-Rad, Hercules, CA, USA). RDW, hemoglobin, and white blood cell (WBC) count were measured as part of the automated complete blood count using an ADVIA 2120 (Siemens, Erlangen, Germany). Fasting serum insulin was determined by chemiluminescence (RIA kit, Daiichi, Japan), and insulin resistance was calculated using the homeostasis model assessment of insulin resistance (HOMA-IR) index, using following formula: HOMA-IR = fasting insulin (*μ*U/mL) × fasting plasma glucose (mmol/L)/22.5.

### 2.4. Carotid Artery Intima-Media Thickness (C-IMT)

C-IMT was measured by high-resolution B-mode ultrasonography on a single machine (Aloka, Tokyo, Japan) with a 7.5 MHz linear array transducer by the same investigator throughout the study. The probe scanned the far wall of the middle and distal common carotid artery by a lateral longitudinal projection. IMT was defined as the distance between the lumen-intima interface and media-adventitia interface. The measurement was made at 20 mm proximal to the origin of the right and left carotid bulb using the computer-assisted analyzing system (M'ATH, METRIS Co., Argenteuil, France) in conjunction with the ultrasound exam. The mean value of 99 computer-based points in this region was calculated to be the C-IMT [17]. The carotid atherosclerosis was defined to be present when C-IMT was greater than or equal to 1.0 mm at either side of the carotid artery, assessed by B-mode ultrasound [10, 18, 19]. The C-MT investigator was blinded to the RDW results.

### 2.5. Statistical Analysis

Data were expressed as the mean ± S.D. One way analysis of variance (ANOVA) was used to compare various continuous variables among the groups. Correlation coefficients between C-IMT and various clinical factors were calculated with Pearson's test. Triglycerides, insulin, and HOMA-IR were log-transformed for analysis since they showed skewed distributions. Enter method multiple linear regression analysis was used to assess whether the factors shown to be significantly associated with C-IMT remained significant after adjustment. The association of the RDW with carotid atherosclerosis was further explored by categorizing the RDW into tertiles and using the first tertile as the reference. Adjusted odds ratios (ORs) and corresponding 95% confidence intervals were estimated with the use of multivariate logistic regression analysis models. Statistical analyses were carried out using SPSS for Windows 20.0 (SPSS Inc., Chicago, IL, USA). *p* values <0.05 were considered statistically significant.

## 3. Results

### 3.1. Baseline Clinical Characteristics of Subjects

Clinical and biochemical characteristics of the study participants are summarized in Table 1. The participants were stratified into three groups according to RDW. Several parameters showed significant differences among the groups. Participants in the highest tertile RDW were older, more likely to be smokers, obese, and had higher blood pressure and longer duration of diabetes compared to subjects in the lowest tertile.

**Figure 1:**
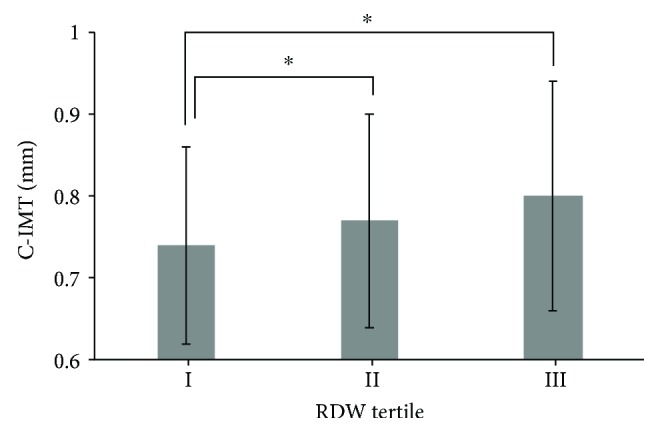
Relationship between C-IMT and RDW tertiles in type 2 diabetes.The C-IMT values significantly increased across RDW tertiles. ^∗^*p* < 0.01 versus the first tertile.

C-IMT values were 0.740 ± 0.120 mm, 0.772 ± 0.138 mm, and 0.795 ± 0.139 mm, in the order of first, second, and third RDW tertiles, respectively, and ANOVA showed significant differences in C-IMT values between tertiles 1 and 2 and tertiles 2 and 3 (Figure 1).

### 3.2. Relationship between RDW and Various Metabolic Parameters

Correlation analyses revealed that C-IMT significantly correlated with age, male gender, BMI, SBP, DBP, insulin, HOMA-IR, smoking, and RDW. In multiple linear regression analysis, RDW was associated with C-IMT after control for covariates (Table 2).

The association between RDW and carotid atherosclerosis was further explored by categorizing RDW into three groups and using the first group as the reference. Compared to subjects at the lowest tertile of RDW, those at the 2nd and 3rd tertile of RDW were at a significantly higher risk of having C-IMT ≥ 1.0 mm (OR 1.68 and 2.12, resp.), after adjusting for age and sex. Those at the highest tertile RDW were 1.95 times more likely to have C-IMT ≥ 1.0 mm after adjusting for various factors related to atherosclerosis, including blood pressure, smoking, BMI, use of statin, and lipid and glucose parameters (Table 3).

## 4. Discussion

In this study, we investigated the relationship between RDW and C-IMT as a marker of subclinical atherosclerosis in people with type 2 diabetes without a history of cardiovascular or cerebrovascular diseases. A positive association was identified between RDW and C-IMT, and it remained significant after adjusting for other cardiovascular risk factors. Participants with highest tertile RDW were over twice more likely to have C-IMT greater than or equal to 1.0 mm compared to those with the lowest tertile. To the best of our knowledge, this is the first study to demonstrate a correlation between RDW and C-IMT in people with type 2 diabetes.

Our results showed that elevated RDW is associated with age, prevalence of hypertension, BMI, SBP, and smoking in people with type 2 diabetes mellitus. These findings are consistent with those of previous studies [20–22]. On the basis of these studies, we believe that atherosclerotic risk factors might be closely related to RDW.

Recently, several studies have demonstrated the association between RDW and increased cardiovascular events and mortality [3–6]. However, there are only few reports on the relationship between RDW levels and subclinical atherosclerosis. Moreover, the results have been inconsistent. Some studies failed to show any association between RDW and C-IMT [11, 12]. In contrast, Wen et al. reported that high RDW levels were associated with C-IMT and carotid plaque in hypertensive people [13]. Other studies identified significant associations between RDW levels and C-IMT in people with ischemic stroke and high-risk group of angina [14, 15]. Recently, Söderholm et al. also reported an association between RDW and C-IMT in the general population [16]. In agreements with several prior studies, the present study showed that RDW is correlated with C-IMT after adjusting other cardiovascular risk factors in people with type 2 diabetes as well.

Although C-IMT measurement can be done without concerns for radiation exposure or radiocontrast dye-induced nephropathy compared with other diagnostic tools for atherosclerosis, such as cardiac computerized tomography or brain magnetic resonance angiography, it is expensive and many clinics are not equipped with the ultrasound. Therefore, along with other well-known risk factors for atherosclerosis, an increased RDW could be considered another marker for atherosclerosis in people with type 2 diabetes without CVD.

Although the exact pathophysiological mechanism has not been clarified, it may be linked to oxidative stress and inflammation. Chronic inflammation and oxidative stress play important roles in arterial atherosclerosis [23–27]. Oxidative stress directly damages erythrocytes and leads to shortened erythrocyte survival, resulting in elevated RDW [28]. In a study of 786 disabled community-dwelling elderly women, the antioxidant, selenium, is independently associated with RDW, and this study also suggested that oxidative stress may be a potential underlying biological mechanism for increased RDW [29]. In addition, inflammation might contribute to an increased RDW. Recent studies also supported the association of anisocytosis and inflammatory markers [30]. However, we did not investigate oxidative stress and inflammatory markers, and the precise relationship between them could not be evaluated in this study.

Our study had several limitations. First, owing to the cross-sectional design, we were unable to determine whether there was a causal relationship between RDW and C-IMT in people with type 2 diabetes. Second, our study population was relatively small. A prospective, larger population studies are needed to confirm the relationship, and they may also provide an RDW cutoff point for a clinically significant carotid atherosclerosis or a need for further evaluation. Also, carotid plaque volume has been shown to be a better predictor of cardiovascular risk compared with intima-media thickness [31], and it should be assessed in the future study, Finally, participants in the current study were enrolled in a single urban hospital, resulting in the possibility of selection bias. Although our study had limitations in generalizing the results, it is the first study to clarify the association between RDW and C-IMT in people with type 2 diabetes.

## 5. Conclusions

We found a significant association between RDW and C-IMT in people with type 2 diabetes without a history of cardiovascular or cerebrovascular diseases. Increased RDW might be a risk factor for subclinical atherosclerosis in people with type 2 diabetes.

## Figures and Tables

**Table 1:** Clinical characteristics of subjects according to RDW tertiles.

	I (10.9–12.1%)	II (12.2–12.6%)	III (12.7–16.0%)	*p* value
*N*	158	157	154	
Male (%)	105 (66.5)	88 (56.1)	100 (64.9)	0.22
Age (years)	54.29 ± 10.57	56.88 ± 11.96	59.92 ± 12.44	<0.01
Diabetes duration (years)	6.78 ± 6.81	7.25 ± 7.57	7.61 ± 7.43	<0.01
BMI (kg/m^2^)	24.15 ± 2.77	24.58 ± 3.41	25.28 ± 3.57	0.01
SBP (mmHg)	124.18 ± 13.31	127.03 ± 14.19	128.07 ± 14.54	0.04
DBP (mmHg)	77.08 ± 9.06	77.66 ± 8.27	77.48 ± 8.85	0.37
FPG (mmol/L)	9.09 ± 3.94	9.16 ± 3.73	9.57 ± 4.09	0.16
HbA1c (%)	8.62 ± 2.24	8.63 ± 2.43	8.69 ± 2.23	0.39
HbA1c (mmol/mol)	70.7 ± 1.0	70.8 ± 3.1	71.5 ± 0.9	0.41
TC (mmol/L)	4.46 ± 1.06	4.37 ± 1.09	4.55 ± 1.11	0.33
TG (mmol/L)	1.55 (1.15–2.19)	1.36 (1.06–1.99)	1.32 (1.01–1.72)	0.07
LDL-C (mmol/L)	2.55 ± 1.03	2.56 ± 0.93	2.57 ± 0.98	0.76
HDL-C (mmol/L)	1.13 ± 0.33	1.14 ± 0.33	1.15 ± 0.31	0.85
Insulin (mIU/dL)	6.0 (3.0–9.2)	6.75 (2.5–9.8)	7.6 (2.8–11.4)	0.31
HOMA-IR	2.50 (1.07–3.87)	2.31 (1.03-4.25)	2.32 (1.04–4.56)	0.23
Hemoglobin (g/dL)	14.28 ± 1.50	13.78 ± 1.61	13.55 ± 1.63	<0.01
WBC (10^3^/mL)	6.76 ± 1.78	6.84 ± 1.88	6.93 ± 1.63	0.22
Antidiabetic medication (%)				
Thiazolidinedione	45 (28.5)	46 (29.3)	41 (26.6)	0.45
Metformin	134 (84.8)	139 (82.5)	128 (83.1)	0.67
Sulfonylurea	80 (50.6)	76 (48.4)	84 (54.5)	0.51
DPP-IV inhibitor	39 (24.7)	37 (23.6)	39 (25.3)	0.20
Insulin	21 (13.3)	25 (15.9)	22(14.3)	0.37
Smoking (%)	50 (31.6)	53 (33.8)	69 (44.8)	0.02
Hypertension (%)	75 (47.4)	83 (52.9)	91 (59.1)	<0.01
Statin use (%)	92 (58.2)	94 (59.9)	99 (64.3)	0.42

Data are represented as the mean ± SD, number (percentage), or median (interquartile range). RDW: red blood cell distribution width; BMI: body mass index; SBP: systolic blood pressure; DBP: diastolic blood pressure; FPG: fasting plasma glucose; HbA1c: hemoglobin A1c; TC: total cholesterol; TG: triglyceride; LDL-C: low-density lipoprotein cholesterol; HDL-C: high-density lipoprotein cholesterol; HOMA-IR: homeostasis model assessment of insulin resistance; WBC: white blood cell.

**Table 2:** Correlations and multiple regression of risk factors associated with C-IMT.

	*γ*	*p* value	*β*	*p* value
Age	0.497	<0.01	0.228	<0.001
Sex (M versus F)	0.229	<0.01	0.191	<0.001
DM duration (yrs)	0.216	<0.01	0.087	0.108
BMI (kg/m^2^)	0.089	0.047	0.039	0.459
SBP (mmHg)	0.196	0.031	0.135	0.041
DBP (mmHg)	0.107	0.036	0.057	0.323
FPG (mmol/L)	0.013	0.590	—	—
HbA1c (%)	0.027	0.565	—	—
TC (mmol/L)	0.050	0.192	—	—
TG (mmol/L)	0.105	0.074	—	—
LDL-C (mmol/L)	0.082	0.322	—	—
HDL-C (mmol/L)	−0.062	0.190	—	—
Insulin (mIU/dL)	0.092	0.061	—	—
HOMA-IR	0.125	0.020	0.100	0.042
RDW (%)	0.162	<0.01	0.112	0.030
Smoking (smokers versus nonsmokers)	0.173	<0.01	0.131	0.015

C-IMT: carotid intima-media thickness; M: male; F: female; DM: diabetes mellitus; BMI: body mass index; SBP: systolic blood pressure; DBP: diastolic blood pressure; FPG: fasting plasma glucose; HbA1c: hemoglobin A1c; TC: total cholesterol; TG: triglyceride; LDL-C: low-density lipoprotein cholesterol; HDL-C: high-density lipoprotein cholesterol; HOMA-IR: homeostasis model assessment of insulin resistance; RDW: red blood cell distribution width. Continuous variables with skewed distributions (TG, insulin, and HOMA-IR) were log-transformed for analysis.

**Table 3:** Odds ratios and 95% confidence intervals for increased carotid IMT according to RDW.

	RDW tertile			
	I	II	III	*p* for trend
OR (model 1)	1.00	1.89 (1.10–3.25)	2.77 (1.63–4.70)	<0.01
OR (model 2)	1.00	1.68 (1.03–2.80)	2.12 (1.18–4.23)	<0.01
OR (model 3)	1.00	1.28 (0.87–2.01)	1.95 (1.08–3.52)	0.03

RDW: red blood cell distribution width; OR: odds ratio. Carotid atherosclerosis was defined as C-IMT ≥ 1.0 mm. Model 1: unadjusted. Model 2: adjusted for age and sex. Model 3: adjusted for age, sex, DM duration, BMI, SBP, DBP, TG, HDL-C, LDL-C, HOMA-IR, and smoking.
